# Our Baby: For Mothers and Nurses

**Published:** 1891-06

**Authors:** 


*Our Baby: for Mothers and Nurses.* By Mrs. LANGTON HEWER. Bristol: JOHN WRIGHT & Co. 1891.

It is a pleasure to be able to say that this is an admirable book to put into the hands of one who is about to, or has just, become a mother, or of one who has the charge of young children. It abounds with practical information, clearly and briefly expressed.

Chapter II., entitled "Is Baby All Right?" is the one we like least in the book. It contains scarcely anything that the young mother need know, but much that would give her many an unnecessary fright. The chapters on "Baby's Illnesses" and "Medicines for Baby" are full of the dangers which usually accompany directions such as are there given, and this notwithstanding the caution against treating the disease and not the child. But as, in spite of some peculiar punctuation, it seems that these were written by a "London" physician, it is perhaps irreverent to offer this criticism. To him, we suppose, must also be credited the display of erudition in the closing sentence of the book. As the volume is intended for popular use, his excellent aphorism would have been none the less forcible had he given it in English, so that all mothers and nurses might have understood it.

Mrs. Hewer is not quite logical in her observations about Nature's indications for weaning, and she pays too little regard to the fact that many mothers with the best intentions are wholly incapable, or only very partially capable, of nursing their offspring.

But these slight blemishes do not detract from the general merits of Mrs. Hewer's excellent little book. Perhaps her most useful sentence is this: "Infants' stomachs need rest." Printed on a card, and kept prominently before a distracted mother or a fussy nurse, it might save an unfortunate baby many an uncomfortable pang.

We were specially pleased with the chapter on "Baby's Nurse."

